# Prevalence and Associations of General Practice Registrars’ Management of Impetigo: A Cross-Sectional Analysis From the Registrar Clinical Encounters in Training (ReCEnT) Study

**DOI:** 10.5826/dpc.1002a43

**Published:** 2020-04-03

**Authors:** Hilary Gorges, Clare Heal, Mieke van Driel, Amanda Tapley, Joshua Davis, Andrew Davey, Elizabeth Holliday, Jean Ball, Nashwa Najib, Neil Spike, Kristen Fitzgerald, Parker Magin

**Affiliations:** 1 Mackay Clinical School, College of Medicine and Dentistry, James Cook University, Mackay, Australia; 2 Primary Care & General Practice, Faculty of Medicine, University of Queensland, Australia; 3 GP Synergy, Australia; 4 Global and Tropical Health Division, Menzies School of Health Research School of Medicine, Darwin, Australia; 5 Biostatistics, School of Medicine and Public Health, University of Newcastle, Callaghan, Australia; 6 Clinical Research Design and Statistics, Hunter Medical Research Institute, New Lambston Heights, Australia; 7 Department of General Practice, The University of Melbourne, Notting Hill, Australia; 8 General Practice Training Tasmania

**Keywords:** impetigo, prevalence, bacterial, primary care, skin infection

## Abstract

**Background:**

Impetigo is a mild bacterial skin infection of childhood that is usually managed empirically in primary care.

**Objective:**

To establish the prevalence and associations of impetigo in general practice (GP) registrars’ consultations.

**Methods:**

Cross-sectional analysis of the Registrar Clinical Encounters in Training (ReCEnT) study data.

**Results:**

Impetigo was managed in 0.24% of problems and 0.43% of consultations. Patient variables associated with impetigo presentations were younger age and impetigo as a new problem, while patients with non–English-speaking backgrounds were less likely to present with impetigo. Associated registrar variables were being new to the registrar and practicing in outer regional/remote locations. Compared with all other problems/diagnoses, impetigo more often involved information seeking, ordering pathology, and prescription of medication, but less often involved follow-up or referral.

**Conclusions:**

Impetigo accounts for 0.43 per 100 GP registrar consultations in Australia. Association with outer regional/remote areas may reflect climate and socioeconomic factors that predispose to impetigo. Associated pathology requests may reflect a lack of confidence in GP registrars’ management of impetigo. Cultural differences may exist regarding health-seeking behavior relating to impetigo.

**What is known about the topic?**

Impetigo is a bacterial skin infection, most prevalent in remote Aboriginal communities, with implications for antimicrobial stewardship.

**What does this paper add?**

Prevalence of impetigo and associated characteristics in the Australian population, outside of remote Aboriginal communities.

## Introduction

Impetigo is a common skin infection [1,2] caused by *Staphylococcus aureus* or *Streptococcus pyogenes* [2,3]. It is often seen in childhood and is associated with poverty and tropical environments [2] and is most prevalent in remote indigenous Australian children [2]. As a mild disease with good prognosis and potential for self-resolution [3], impetigo is managed in primary care [3,4], but because of its significant disease burden and highly contagious nature [2,3], empirical antibiotic treatment is recommended. Skin swabs for bacterial culture are reserved for severe/recurrent disease or empirical treatment failure [5,6]. Thus the management of impetigo has considerable implications for antibiotic stewardship. Previous analysis from the Registrar Clinical Encounters in Training (ReCEnT) study [7] raised questions regarding interpretation of Australian guidelines for impetigo management and the subsequent implications for antibiotic stewardship. Characterizing the prevalence of impetigo and its associations is important to optimize and explore management that promotes antimicrobial stewardship.

Skin conditions are among the most common problems in Australian general practice (GP), managed in 17.4 per 100 encounters, and they account for 11.3% of problems [8]. Despite this frequency, studies have shown that registrars find skin conditions challenging and are not adequately prepared for the dermatology burden in the community [9]. Impetigo prevalence in global communities has been measured extensively, and while it is highest in remote Aboriginal and Torres Strait Islander populations, with a median prevalence of 44.5% (interquartile range [IQR] 34%–49.2%), there is a lack of data for non-Aboriginal and Torres Strait Islander Australian populations [2].

The prevalence of impetigo presenting in the general population in Australia is unknown, and its associations are unexplored. The aim of this study is to investigate the prevalence of impetigo presenting to Australian GP registrars, and the associations and outcomes of these consultations.

## Methods

### ReCEnT Project

Data were analyzed from the multisite cohort study of Australian GP registrars’ clinical practice, detailed methodology of which is described elsewhere [10].

#### Outcome Factor

The outcome factor was a problem/diagnosis being “impetigo,” defined by the International Classification of Primary Care, second edition classification system [11] as code S84.

Independent variables were related to registrar, patient, practice, and consultation.

#### Registrar Variables

Registrar variables were age, sex, training term, place of medical qualification (Australia/international), full-time/part-time, and worked at the practice previously.

#### Patient Variables

Patient variables were age, sex, Aboriginal/Torres Strait Islander, non–English-speaking background (NESB), problem/diagnosis was new, new to the practice, and new to the registrar.

#### Practice Variables

Practice variables were training “region,” rurality/urbanity, practice size (number of GPs), socioeconomic status of area, and routine bulk-billing. Postal codes defined rurality of location using Australian Standard Geographical Classification-Remoteness Area classification [12] and the location’s Socioeconomic Index for Area Relative Index of Disadvantage [13].

#### Consultation and Educational Variables

Consultation variables included duration, number of diagnoses/problems, pathology/imaging ordered, specialist referral made, follow-up organized, and medication prescribed. Educational variables included seeking information or assistance and generating learning goals.

### Statistical Analysis

We conducted cross-sectional analysis of data from the ReCEnT cohort study performed on 16 rounds of 6-monthly collected data from 2010–2017, with analysis at the individual problem/diagnosis level.

The proportion of registrars’ impetigo problems/diagnoses and the proportion of impetigo consultations were calculated, with 95% confidence intervals (CIs).

#### Impetigo vs Other Problems/Diagnoses

To test associations of a problem/diagnosis being impetigo, simple and multiple logistic regression analyses were used within the generalized estimating equations framework, accounting for clustering of patients within registrars. All variables with a P value <0.20 in univariate analysis were included in the multiple regression model. Covariates with P > 0.2 in the multivariable model were tested for removal. Covariates were removed if this did not substantively change the remaining coefficients in the model.

To examine 3 separate issues within our research question, 3 models were built, each with the dependent variable “impetigo problem/diagnosis”:

1. To examine associations of a problem/diagnosis being impetigo (compared with other problems/diagnoses), patient, practice and registrar independent variables were entered in a regression model.
2. To examine in-consultation differences of an impetigo problem/diagnosis compared with other problems/diagnoses, the above variables were entered in a model along with consultation duration, information/assistance accessed by registrar, and number of problems/diagnoses dealt with in consultation.
3. To examine whether actions from managing impetigo differ from those managing other problems/diagnoses, all variables entered in the previous 2 models were entered in a model along with learning goals generated, follow-up organized, specialist referrals made, and pathology and imaging ordered.

Statistical analyses were programmed using STATA 14.0 and SAS v9.4. P values <0.05 were considered statistically significant.

### Ethics Approval

The University of Newcastle Human Research Ethics Committee approved the study (Reference H-2009-0323).

## Results

A total of 1,741 registrars (response rate 96%) contributed 377,980 patient problems over 214,888 consultations. Impetigo was diagnosed in 915 consultations, accounting for 0.24% of all problems and managed in 0.43 per 100 consultations.

### Characteristics Associated With an Impetigo Diagnosis

On multivariable analysis, younger patients (age 0–14, compared to 15–34: odds ratio [OR] 7.14, 95% CI 5.75–8.86, P < 0.001) and patients new to the registrar (OR 1.33, 95% CI 1.11–1.60, P = 0.002) were more likely to present with impetigo (Table 1). NESB patients were less likely to present with impetigo (OR 0.57, 95% CI 0.36–0.89, P = 0.015). Registrars working in outer regional/remote/very remote areas (OR 1.66, compared to major cities: 95% CI 1.11–2.48, P = 0.014) and male registrars (OR 1.20, 95% CI 1.45–1.01, P = 0.038) were more likely to see patients with impetigo.

### What Happens Differently During Impetigo Consultations?

Impetigo consultations were significantly shorter in duration (14 vs 19 minutes on unadjusted analysis and OR 0.94 per additional minute, 95% CI 0.93–0.96, P < 0.001 on multivariable analysis) and the registrars were more likely to seek help (OR 2.53, 95% CI 2.13–3.02, P < 0.001) (Table 1). Of those who sought help from supervisors, the highest proportion were first-term registrars (70.7%) (Table 2). The most frequent resource type used was electronic; the most common was Therapeutic Guidelines (61.65%) (Tables 2 and 3). Impetigo was more likely to present as a new, rather than existing, problem (OR 2.19, 95% CI 1.74–2.76, P < 0.001).

### What Outcomes Are Different in Impetigo Consultations?

Impetigo consultations were significantly more likely to involve pathology being ordered (OR 2.43, 95% CI 1.93–3.06, P < 0.001) (Table 1), the majority being skin swabs for bacterial culture (47%) (Table 4). Impetigo consultations were more likely to involve medication prescription (OR 12.8, 95% CI 9.34–17.5, P < 0.001) but less likely to result in follow-up (OR 0.71, 95% CI 0.59–0.86, P = 0.001) or referral (OR 0.11, 95% CI 0.04–0.29, P < 0.0001). Of impetigo consultations, 83% did not result in generation of a learning goal.

## Discussion

### Prevalence

Impetigo accounted for 0.24% of problems seen by Australian GP registrars and was managed in 0.43 per 100 consultations. Finding comparable data regarding the prevalence of impetigo is difficult. The BEACH study grouped impetigo with other “skin problems,” which were seen in 17.4 per 100 consultations [8]. In Aboriginal and Torres Strait Islander communities, impetigo encounters have been recorded as high as 7.5 per 100 consultations [14]. In a Dutch study, yearly impetigo incidence was 4 per 100 patients aged <18 years; however, the proportion of consultations was not measured [15]. Impetigo prevalence in global communities has been measured more extensively, although the majority of data was collected before 2010, with only 3 studies from 2010–2015 (3%) [2]. Impetigo prevalence is highest in remote Aboriginal and Torres Strait Islander populations with a median prevalence of 44.5% (IQR 34%–49.2%) [2]. A Canadian study of 2 First Nation communities found impetigo prevalence between 1% and 4.2% [16]; in comparison, the median prevalence of impetigo in Africa is 7% (IQR 4.1%–12.3%) [2] and 0.75% in Japan [17]. There is a paucity of prevalence data for North America, China, most European countries and non-Aboriginal Australian populations [2,18].

### Impetigo Diagnosis Characteristics

Registrars working in outer regional/remote areas were more likely to see patients with impetigo. Impetigo is most prevalent in poorly resourced areas and tropical/arid climates [2]; the regional differences seen in our study may reflect lower income level and more tropical/arid climates [12,19] in Australian outer regional/remote areas.

Most patients with impetigo were <14 years, consistent with impetigo being a childhood disease [1,3]. Registrars see younger patients than do established GPs [8,20]; of registrar consultations, 17% involve patients <14 years [20,21] compared with 12% for established GPs [8]. Registrars are also more likely to see new patients, new problems, and acute illnesses [8], which may result in an elevated proportion of impetigo consultations in our study compared with mainstream GPs.

Patients being more likely to present to the registrar with impetigo as a new problem or for the first time reflects impetigo’s acute nature. Patients may be more willing to see registrars for acute issues or perceived minor problems [22]. Consultations for acute conditions are also booked on shorter notice, with the first doctor available, who is usually the registrar [9].

NESB patients were less likely to present with impetigo. One-tenth of GP patients are from NESB [8] and 21% of Australians speak a language other than English at home [23]. Our finding may reflect cultural practices that predispose patients to impetigo; also as impetigo is a mild condition with the potential for self-resolution, this may reflect different cultural approaches to health-seeking behavior regarding impetigo.

### Differences in Impetigo Consultations

Impetigo consultations were shorter; this is expected as impetigo is a common condition with a clinical diagnosis, systemically well patients [3,4], and clear guidelines available [6].

Registrars are more likely to seek information/assistance for impetigo despite its being a mild disease. First-term registrars were more likely to seek help from their supervisor than those in advanced terms, which may reflect initial inadequate preparation for the dermatological disease burden in the community [9], with confidence improving with experience. The most commonly used resources were electronic (Table 2); the Therapeutic Guidelines [6] were most often used (Table 3). In impetigo consultations the majority of registrars (83%) did not generate learning goals, patients were mostly children, and medication was usually prescribed; therefore, registrars may have used this reference for dose checking rather than guidance on diagnosis/management.

### Different Outcomes in Impetigo Consultations

Impetigo consultations were more likely to involve pathology being ordered, the majority being skin swabs for bacterial culture (Table 4), and medication prescription. As guidelines advise empirical treatment for impetigo, reserving skin swabs for severe, resistant, or recurrent disease [6], this may demonstrate lack of confidence with treating dermatological conditions. Follow-up and referrals were less likely in impetigo consultations, reflecting the mild nature of the disease and preferred management in primary care [3,4].

### Strengths and Limitations

The strengths of this study are a large sample size of GP registrars with a high response rate from across Australia, including major cities and remote areas. A limitation of the analysis and interpretation of the data is the absence of information regarding the severity of impetigo. We are unable to account for this variable in our interpretation, and low numbers of Aboriginal and Torres Strait Islander patients (4%) means we were underpowered to analyze this variable.

## Conclusions

Impetigo accounts for 0.43 per 100 consultations seen by Australian GP registrars. It is more common in outer regional/remote/very remote areas and usually presents as a new problem. NESB patients are less likely to present with impetigo, which may reflect different cultural approaches to the management of a mild, potentially self-limiting condition. Impetigo was associated with pathology being ordered, which may reflect a lack of confidence in Australian GP registrars when dealing with this presentation.

## Figures and Tables

**Table 1:** Patient, Registrar, and Consultation Characteristics Associated With Impetigo

Variable	Class	Univariate		Adjusted	
		OR (95% CI)	P	OR (95% CI)	P
**Patient variables**					
Patient age group	0–14 years	7.97 (6.64, 9.57)	<0.0001	7.14 (5.75, 8.86)	<0.0001
	35–64 years	0.38 (0.29, 0.50)	<0.0001	0.38 (0.28, 0.52)	<0.0001
	65+ years	0.12 (0.07, 0.20)	<0.0001	0.12 (0.07, 0.24)	<0.0001
Patient sex	Female	0.66 (0.57, 0.76)	<0.0001	0.87 (0.74, 1.02)	0.0795
Aboriginal or Torres Strait Islander	Yes	2.66 (1.78, 3.98)	<0.0001	1.48 (0.96, 2.28)	0.0758
Non–English-speaking background	Yes	0.40 (0.27, 0.58)	<0.0001	0.57 (0.36, 0.89)	0.0145
Patient/practice status	New to practice	1.90 (1.44, 2.50)	<0.0001	0.93 (0.67, 1.29)	0.6588
	New to registrar	2.11 (1.80, 2.48)	<0.0001	1.33 (1.11, 1.60)	0.0018
**Registrar variables**					
Registrar sex	Female	0.75 (0.64, 0.87)	0.0003	0.83 (0.69, 0.99)	0.0382
Worked at practice previously	Yes	0.85 (0.70, 1.03)	0.0992	0.82 (0.66, 1.02)	0.0758
Registrar age		1.01 (1.00, 1.02)	0.1100	1.00 (0.98, 1.01)	0.7376
**Practice variables**					
Practice routinely bulk bills	Yes	0.66 (0.53, 0.81)	<0.0001	0.78 (0.60, 1.01)	0.0622
Rurality	Inner regional	1.32 (1.10, 1.58)	0.0024	1.14 (0.91, 1.43)	0.2516
	Outer regional remote	1.41 (1.13, 1.77)	0.0029	1.66 (1.11, 2.48)	0.0139
Socioeconomic Index for Area		1.03 (1.00, 1.05)	0.0587	1.02 (0.98, 1.06)	0.2831
Region	Region 2	0.40 (0.28, 0.57)	<0.0001	0.37 (0.24, 0.56)	<0.0001
	Region 3	0.84 (0.66, 1.07)	0.1525	0.66 (0.48, 0.90)	0.0080
	Region 4	0.65 (0.54, 0.77)	<0.0001	0.54 (0.42, 0.68)	<0.0001
	Region 5	1.47 (1.00, 2.16)	0.0475	0.79 (0.45, 1.39)	0.4087
	Region 6	0.49 (0.34, 0.72)	0.0002	0.47 (0.29, 0.78)	0.0032
**Consultation variables**					
New problem seen	Yes	4.14 (3.36, 5.11)	<0.0001	2.19 (1.74, 2.76)	<0.0001
Sought help any source	Yes	2.68 (2.32, 3.11)	<0.0001	2.53 (2.13, 3.02)	<0.0001
Consultation duration		0.93 (0.92, 0.94)	<0.0001	0.94 (0.93, 0.96)	<0.0001
No. of problems		0.44 (0.40, 0.49)	<0.0001	0.95 (0.84, 1.07)	0.4079
**Consultation outcome variables**					
Pathology ordered	Yes	1.13 (0.95, 1.35)	0.1705	2.43 (1.93, 3.06)	<0.0001
Follow-up ordered	Yes	0.83 (0.72, 0.96)	0.0137	0.71 (0.59, 0.86)	0.0004
Referral ordered	Yes	0.03 (0.01, 0.08)	<0.0001	0.11 (0.04, 0.29)	<0.0001
Medication prescribed	Yes	15.7 (12.1, 20.3)	<0.0001	12.8 (9.34, 17.5)	<0.0001

CI = confidence interval; OR = odds ratio.

**Table 2:** Resource Type Used for Impetigo Consultations Presented by Training Term

Resource Type Used in Impetigo Consultation	Term 1 Frequency (%)	Term 2 Frequency (%)	Term 3 Frequency (%)	Total
Electronic	113 (52.6)	54 (25.1)	48 (22.3)	215
Supervisor	65 (70.7)	21 (22.8)	6 (6.5)	92
Book	13 (72.2)	3 (16.7)	2 (11.1)	18
Specialist	0 (0)	2 (66.7)	1 (3.3)	3
Other	5 (55.6)	2 (22.2)	2 (22.2)	9

**Table 3:** Electronic Resources Used for Impetigo Consultations

Resource	Frequency (%)
Therapeutic Guidelines	127 (61.65)
Royal Children’s Hospital Guidelines	22 (10.68)
DermNet NZ	17 (8.25)
Australian Medicines Handbook	11 (5.34)
Monthly Index of Medical Specialties (MIMS online)	6 (2.91)
Murtagh’s General Practice	4 (1.94)
Other	19 (9.2)
Total	206

**Table 4:** Pathology Ordered for Impetigo

Pathology request	Frequency (%)
Skin swab MC&S	96 (47)
Swab MC&S	57 (28)
Nose swab MC&S	16 (7.9)
Herpes simplex culture	11 (5.4)
Viral swab MC&S	4 (2)
MC&S	3 (1.5)
Other	16 (7.9)
Total	203

MC&S = microscopy, culture and sensitivity.
